# Association of High Dietary Acid Load With the Risk of Cancer: A Systematic Review and Meta-Analysis of Observational Studies

**DOI:** 10.3389/fnut.2022.816797

**Published:** 2022-03-28

**Authors:** Majid Keramati, Sorayya Kheirouri, Vali Musazadeh, Mohammad Alizadeh

**Affiliations:** ^1^Student Research Committee, Tabriz University of Medical Sciences, Tabriz, Iran; ^2^Faculty of Nutrition and Food Sciences, Tabriz University of Medical Sciences, Tabriz, Iran

**Keywords:** dietary acid load, cancer, systematic review, meta-analysis, observational studies

## Abstract

**Objective:**

This study aimed to determine the relationship between the high dietary acid load (DAL) and the risk of cancer.

**Methods:**

Five databases of PubMed, Web of Sciences, Scopus, Cochrane Library, and Google Scholar was searched to elicit original studies on humans, up to June 2021. Quality of the articles, risk of bias, and heterogeneity were assessed. A random-effects meta-analysis model was applied to estimate pooled effect size with a 95% confidence interval. Sensitivity analysis was performed using a fixed-effects model. Subgroup analyses were carried out based on gender, age, type of cancer, and type of DAL assessment indicator.

**Results:**

Seventeen effect sizes from 10 articles were included in the analysis. Overall, individuals with the highest DAL were associated with a 66% increased risk of cancer compared to those with the lowest DAL (*p* < 0.001]. The risk of cancer increased 41% (*p* < 0.001) and 53% (*p* = 0.03) by high PRAL and NEAP, respectively. High DAL was associated with 32% (*p* < 0.001) and 79% (*p* < 0.001) increased risk of breast and colorectal cancers, respectively. High DAL was associated with 32% (*p* = 0.001) and 76% (*p* = 0.007) increased risk of cancer incident in women and men, respectively. The risk of cancer incident increased 35% (*p* < 0.001) and 49% (*p* < 0.001) at age ≤ and > of 50, respectively.

**Conclusion:**

High DAL may be associated with a higher risk of cancer incidence not only in the whole studied population but also across cancer types, both genders, both DAL assessment indicators, and also among both high- and low-risk age groups for cancer.

## Introduction

Cancer is a major burden of disease and health concern worldwide. It is the second leading cause of mortality in many countries (1) and accounting for around 10 million deaths in 2020 (2). It has been well known that lifestyle could influence the risk of cancer (3). An individual's diet is a major modifiable lifestyle-related factor that may be linked to his/her health outcomes. Numerous epidemiological investigations have indicated that diet composition or pattern can contribute to or prevent the development of chronic diseases including cancer (4–6). According to the previous studies, adherence to a plant-based diet with low animal and processed food products may prevent the risk of cancer (7, 8).

It has recently been suggested that diet composition may influence the body's acid-base balance (9). Some dietary components are acidogenic and increase the dietary acid load (DAL). Animal proteins and cereal grains are dietary components that are metabolized to acid precursors and generate acid in the body (10, 11). While, some food ingredients such as fruits and vegetables, due to containing potassium, or dairy products, due to consisting of calcium and magnesium, produce precursors of alkali and may reduce diet-dependent acid load (10, 11).

As the DAL correlates with the urinary acid load, it has been suggested as a simple and useful method to evaluate the acidity of a diet (12). Potential renal acid load (PRAL) and net endogenous acid production (NEAP) are two common established indicators to calculate metabolic acidosis and estimate the DAL from dietary intake data (13, 14). PRAL presents an assessment of the endogenous acid production that exceeds the alkali level produced for certain amounts of food consumed daily. Daily PRAL is a measure that considers the dietary composition of several minerals and proteins (particularly sulfur-containing proteins) and their mean intestinal absorption rate, and the amount of sulfate generated from metabolized proteins. PRAL is calculated using the following formula:

PRAL (mEq/day) = 0.4888 × protein intake (g/day) + 0.0366 × phosphorus (mg/day) – 0.0205 × potassium (mg/day) – 0.0125 × calcium (mg/day) – 0.0263 × magnesium (mg/day)

NEAP is assessed from the ratio of protein and potassium in the diet and calculated using the following formula:

NEAP (mEq/day) = 54.5 × protein (g/day)/potassium (mEq/ day) – 10.2

It has been shown that greater intake of a diet with high acid load may contribute to the increased risk of health conditions such as cardiovascular diseases (15), hypertension (16), chronic kidney disease (17), and diabetes mellitus (18). Multiple investigations have recently studied the association between the DAL and the risk of various cancers (19–24). However, to our knowledge, there has been no comprehensive report summarizing these studies. Therefore, this systematic review and meta-analysis study was implemented to summarize the present studies in order to determine “What is the risk of cancer incidence in adults with high DAL compared to those with low DAL?”

## Methods

This systematic review and meta-analysis study follows the updated 2020 Preferred Reporting Items for Systematic review and Meta-Analysis (PRISMA) guidelines (25). The protocol of the study was registered and approved by the Ethical Committee of Tabriz University of Medical Sciences (IR.TBZMED.REC.1400.560) and is available at: https://ethics.research.ac.ir/IR.TBZMED.REC.1400.560.

### Search Strategy

An extensive systematic search of the literature was performed in electronic databases of PubMed, Web of Sciences, Scopus, Cochrane Library, and Google Scholar up to June 2021, with no publication date restriction. This was supplemented by searching for reference lists and citation tracking of included studies, and relevant reviews. The keywords and medical subject headings (MeSH) terms used for the search were as follows: “acid load OR dietary acid load OR potential renal acid load OR net endogenous acid production” AND “cancer.” The full search method for each database is available in Supplementary Table 1.

The articles from the initial searches were imported into an EndNote software and duplicates were removed. Titles and abstracts of the remained articles were independently screened for potential eligibility by two reviewers (M.K and V.M) and any discrepancy was resolved by discussion or third researcher.

### Inclusion and Exclusion Criteria

The inclusion criteria were as follows: studies that considered the association between DAL and cancer; studies with prospective or retrospective cohort and case-control design; studies that expressed odds ratios (ORs) or hazard ratios (HRs) or relative risks (RRs) beside 95% confidence intervals (CIs) for the association between DAL and cancer. Studies with cross-sectional design, letters, comments, short communications, surveys, environmental, and animal studies were excluded.

### Data Extraction

The required data were extracted from each eligible study by two independent researchers and any disagreement between the two researchers was resolved by discussion or by a third researcher. The extracted information was as follows: Name of the first author, year of publication, country, study design, type of studied cancer, number of participants, mean age and gender of participants, follow-up time for cohort studies, method of food intake assessment, method of DAL assessment, confounding variables, outcomes, and information regarding OR or HR or RR and 95% CI. If a study used both of the PRAL and NEAP indicators for assessing DAL, we considered that study as two separate studies in meta-analysis.

### Quality Assessment

The Newcastle Ottawa (NOS) scale (26) was used to evaluate the quality of the selected studies. Based on this scale, a maximum of 9 scores is allocated to each study as follows: four scores for selection of contributors, two scores for comparability, and three scores for evaluating outcomes in cohort studies and exposure in case-control studies. Studies attaining 9 scores were considered as the highest quality.

### Statistical Analysis

A random-effects model was used to estimate the pooled effect size (d) for comparison of the highest vs. the lowest categories of the DAL and to consider the heterogeneity between the studies (27). The random-effects model was used to estimate the Q-statistics and *I*^2^ values as heterogeneity indices. *I*^2^ value > 50% between-study heterogeneity was considered significant. When between-study heterogeneity was significant, we performed subgroup analyses based on participants' gender, the mean age of the participants, type of cancer, and type of DAL assessment indicator to determine possible sources of heterogeneity. Publication bias was assessed using Egger's and Begg's regression asymmetry test (28). Small study bias, including publication bias, was detected by visually inspecting funnel plots. A trim-and-fill method was used to determine the effect of possible missed studies on the overall effect (29). Sensitivity analysis was performed using a fixed-effects model in which each study was eliminated from the study to evaluate the influence of that study.

STATA version 14.0 was applied to perform statistical analyses. A *p*-value <0.05 was reflecting the statistical significance of all tests.

## Results

### Literature Search

**Figure 1:**
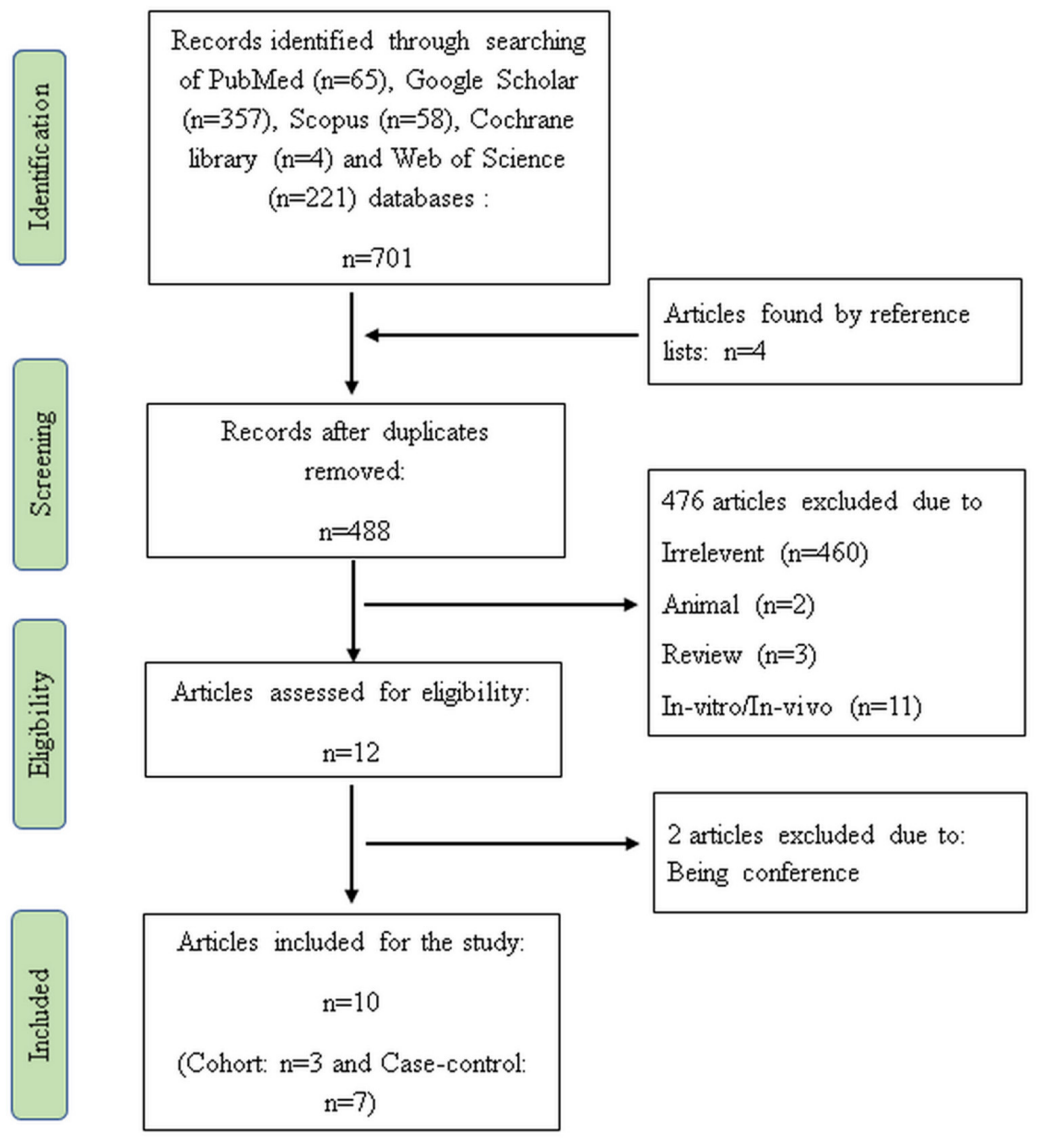
Flow diagram of the study.

In the initial search, 705 articles were detected. After elimination of duplicates (*n* = 217), irrelevant (*n* = 460), animal (*n* = 2), review (*n* = 3), and *in-vitro*/*in-vivo* articles (*n* = 11), 12 publications met the topic and scope of the study during the first screening phase. Two studies were also removed during the second screening phase because these studies were conference. Finally, three cohorts (21, 24, 30) and seven case-control studies (19, 20, 22, 23, 31–33) were comprised in the current systematic review and meta-analysis. Figure 1 shows the flow diagram of the study selection process.

### Characteristics of the Included Studies

Table 1 displays the characteristics of all the included studies. The total number of participants in three cohort studies was 142,228, and in seven case-control studies were 8,060 (2,618 patients with cancer and 5,442 controls). The follow-up period in cohort studies ranged from 7.3 to 8 years. The type of studied cancers were breast (21, 30, 32, 33), colorectal (19, 22), prostate (20), lung (23), pancreas (24), and glioma (31). Most of the studies (*n* = 9) used the food frequency questionnaire (FFQ) tool for assessment of the food intake. In most the papers, effect sizes were adjusted for age (*n* = 10), BMI (*n* = 9), sex (*n* = 3), smoking (*n* = 8), alcohol consumption (*n* = 7), physical activity (*n* = 3), energy intake (*n* = 9), comorbidities (*n* = 4), cancer family history (*n* = 8), menopausal status (*n* = 4), education (*n* = 6), residence (*n* = 3), race (*n* = 3), and other dietary variables (*n* = 6). DAL assessment indicator was PRAL in nine studies and NEAP in seven studies. All cohort and four case-control studies obtained the NOS score of 9 and were of high quality and the score of other studies were 8 (Supplementary Tables 2, 3).

**Table 1:** Characteristics of the included studies.

**References**	**Study design**	**Country**	**Type of cancer**	**Gender**	**Participants (case/control)**	**Mean age (year)**	**Follow-up time**	**Food intake assessment tool**	**DAL assessment indicators**	**Confounders considered in the analysis**	**Outcomes**
Jafari et al. (19)	Case–control	Iran	Colorectal	Both	499 (259/240)	50	-	FFQ	PRAL	Age, comorbidity, CFH, salt intake, physical activity, and Ca supplement	PRAL↑ → risk of CRC and CRA↑
Mehranfar et al. (20)	Case–control	Iran	Prostate	Men	120 (60/60)	Not indicated	-	FFQ	PRAL & NEAP	Age, BMI, TEI, smoking, physical activity, race, job, education, and drug usage	PRAL↑ → risk of prostate cancer↑ NEAP↑ → risk of prostate cancer↑
Mousavi et al. (31)	Case–control	Iran	Glioma	Both	366 (123/243)	42	-	FFQ	NEAP	Age, sex, TEI, marital status, smoking, CFH, physical activity, supplementation, BMI, X-ray exposure, head trauma, allergy, duration of illness, micronutrient intake, and comorbidity	NEAP↑ → developing glioma among adults ↑
Park et al. (21)	Cohort	US and Puerto Rican	Breast	Women	43570	54.5	7.6 y	FFQ	PRAL	Race, education, household income, BMI, physical activity, smoking, alcohol, CFH, breastfeeding, TEI, and parity	PRAL↑ → risk of breast cancer ↑
Ronco et al. (22)	Case–control	Uruguay	Colorectal	Both	3005 (611/2394)	64	-	FFQ	PRAL & NEAP	Age, sex, residence, education, CFH, BMI, smoking, alcohol, TEI, total fiber, micronutrient, and total heterocyclic amines	PRAL ↑ → risk of colorectal cancer ↑ NEAP↑ → risk of colorectal cancer ↑
Ronco et al. (23)	Case–control	Uruguay	Lung	Men	2309 (843/1466)	65	-	FFQ	PRAL & NEAP	Age, residence, CFH, BMI, smoking, alcohol, TEI, total fiber, micronutrient, and total heterocyclic amines	PRAL↑ → was not significantly associated with lung cancer risk NEAP↑ → risk of lung cancer ↑
Ronco et al. (32)	Case–control	Uruguay	Breast	Women	1461 (572/889)	65	-	FFQ	PRAL & NEAP	Age, residence, education, age at menarche, menopausal status, number of live births, age at menopause, CFH, BMI, smoking, alcohol, and TEI	PRAL↑ → risk of breast cancer ↑ NEAP↑ → risk of breast cancer ↑
Safabakhsh et al. (33)	Case–control	Iran	Breast	Women	300 (150/150)	46.5	-	FFQ	PRAL & NEAP	Age at first menarche, BMI, education, marital status, menopause status, socioeconomic status, alcohol, smoking, supplementation, comorbidity, number of Child, breast feeding, CFH, and TEI	PRAL↑ → was not significantly associated with breast cancer risk NEAP↑ → was not significantly associated with breast cancer risk recurrence
Shi et al. (24)	Cohort	US	Pancreatic	Both	95708	64.5	8 y	FFQ & DHQ	PRAL	Age, sex, smoking, diabetes, alcohol, BMI, CFH, TEI, and dietary fiber	PRAL↑ → risk of pancreatic cancer ↑
Wu et al. (30)	Cohort	US	Breast	Women	2950	44	7.3 y	24-h food recall	PRAL & NEAP	Age at diagnosis, race, education, menopausal status at baseline, TEI, alcohol, physical activity, BMI, number of comorbidities, radiotherapy, and chemotherapy.	PRAL↑ → was not significantly associated with breast cancer recurrence NEAP↑ → was not significantly associated with breast cancer recurrence

*DAL, dietary acid load; FFQ, food frequency questionnaire; DHQ, diet history questionnaire; PRAL, potential renal acid load; NEAP, net endogenous acid production; CFH, cancer family history; CRC, colorectal cancer; CRA, colorectal Adenoma; TEI, total energy intake*.

### Risk of Bias Assessment

The methodological characteristics of the included studies are summarized in Supplementary Tables 2, 3. In all of the articles, the most important confounders were controlled in the statistical analysis. In all studies, the selection of controls was done correctly, and in all studies, food intake was assessed by a structured interview.

### Results of Systematic Review and Meta-Analysis

Results of the systematic review showed that of nine studies that investigated the relationship between PRAL and risk of cancer, six studies indicated a positive association. Five out of seven studies found a positive association between NEAP and cancer risk.

**Figure 2:**
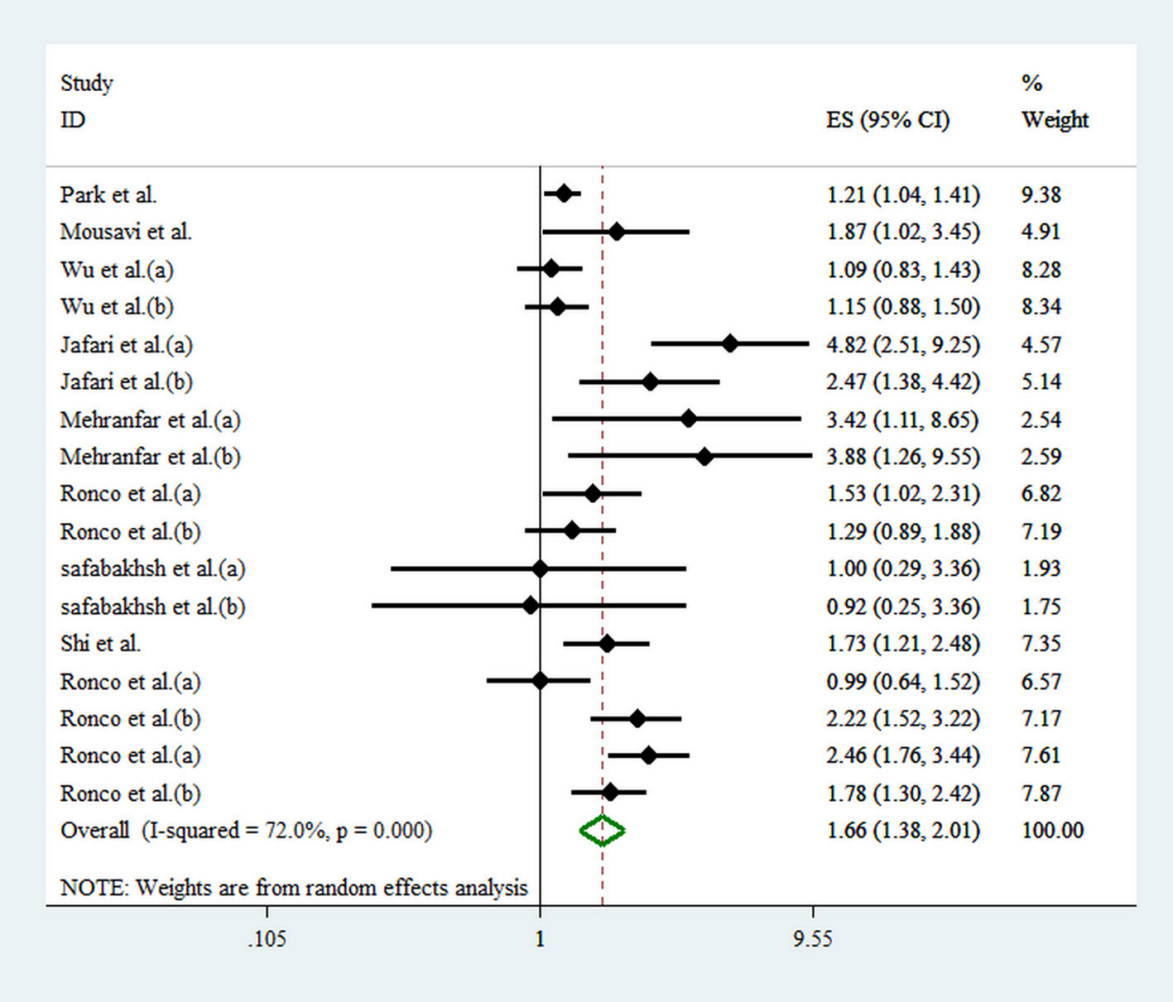
Forest plot for the association between DAL and risk of cancer in a random-effects meta-analysis. ES, effect size; CI, confidence interval.

**Figure 3:**
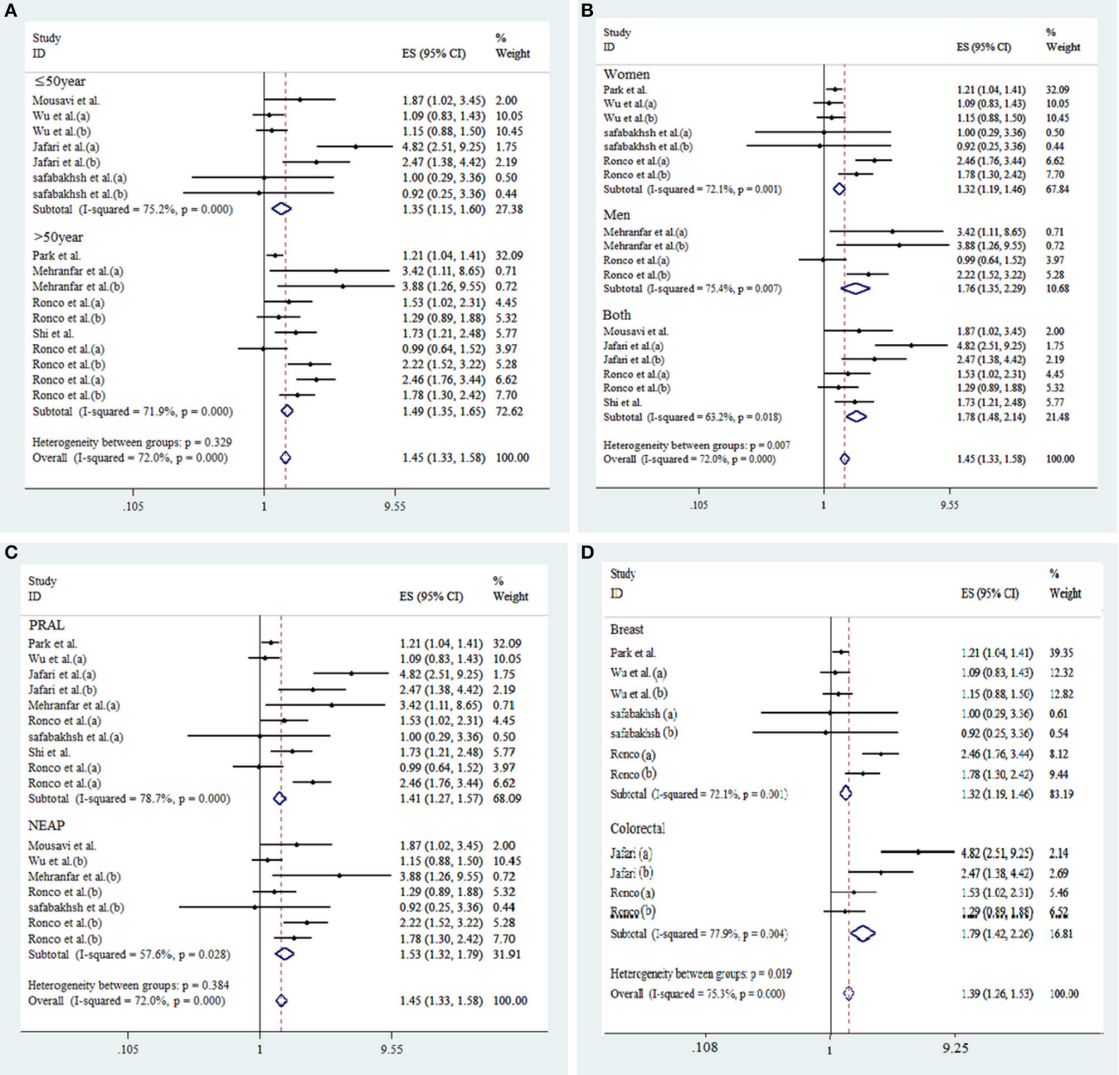
Subgroup analysis for the association between DAL and risk of cancer. Subgroup by age of participants **(A)**, gender **(B)**, type of DAL assessment indicators **(C)**, and type of cancer **(D)**.

Seventeen effect sizes from 10 studies were included in this analysis. Comparing the highest against the lowest DAL, the pooled effect size for the risk of overall cancer was 1.66 (95% CI: 1.38, 2.01; *p* < 0.001), demonstrating a significant positive relationship (Figure 2). A significant heterogeneity between studies was observed (*I*^2^ = 72.0%; *p* < 0.001). As shown in Figure 3, results of subgroup analyses showed that gender, age of the participants, type of cancer, and type of DAL assessment indicator had not any role in the between-study heterogeneity.

As shown in Figure 3, according to stratified analysis, the risk of cancer incidence increased by 41% [d = 1.41 (95% CI: 1.27, 1.57), *p* < 0.001] and 53% [d = 1.53 (95% CI: 1.32, 1.79), *p* = 0.03] by high PRAL and NEAP, respectively. High DAL was associated with 32% [d = 1.32 (95% CI: 1.19, 1.46), *p* < 0.001] and 79% [d = 1.79 (95% CI: 1.42, 2.26), *p* < 0.001] increased risk of breast and colorectal cancers occurrence, respectively. High DAL was associated with 32% [d = 1.32 (95% CI: 1.19, 1.46), *p* = 0.001] and 76% [d = 1.76 (95% CI: 1.35, 2.29), *p* = 0.007] increased risk of cancer incidence in women and men, respectively. The risk of cancer incidence increased by 35% [d = 1.35 (95% CI: 1.15, 1.60), *p* < 0.001] and 49% [d = 1.49 (95% CI: 1.35, 1.65), *p* < 0.001] among people with age ≤ and > of 50, respectively.

### Sensitivity Analyses and Publication Bias

Sensitivity analysis showed that the overall effect size regarding the association between DAL and cancer did not depend on a single study (95% CI: 1.32-2.13). Based on the visual inspection of the funnel plot, we found an asymmetry (Supplementary Figure 4); however, when we did the Egger's and Begg's regression test indicated possible publication bias for the association between DAL and cancer (*p* = 0.038). Therefore, we did the trim-and-fill method and found that adding missing studies did not change the overall effect size [d = 0.36 (95% CI: 0.16-0.56)] (Supplementary Figure 2).

## Discussion

In the present meta-analysis of observational studies, a significant association was observed between higher DAL and the risk of cancer occurrence in the entire population. We found that the risk of cancer increased by 66% in participants with higher DAL compared to the participants with lower DAL. The positive association remained significant across cancer types, both genders, both DAL assessment indicators (PRAL and NEAP), and also among both high- and low-risk age groups for cancer.

It is well known that factors such as sex, age, obesity, energy intake, smoking status, and physical activity level have a role in cancer development. On the other hand, the association of these factors with DAL has been evidenced in numerous researches. It has been shown that DAL has larger effects in the elderly than younger individuals and in women compared with men (34) and elder individuals may be more sensitive to DAL effects compared with younger persons (9). Fatahi et al. showed that the odds of general and abdominal adiposity increased across tertiles of DAL (35). Li et al. have also reported a positive association between high DAL and obesity in the nationally-representative sample of Chinese adults (36). Fatahi et al. in a study on women found a positive association between DAL and energy density (35). Kataya et al. in a study on elderly Japanese women found that high DAL was directly associated with the prevalence of frailty, slowness, and low physical activity (37). Wu et al. did not find any association between DAL and total mortality among never smokers but observed such association among past smokers (38).

Collectively, the above-mentioned factors may contribute to the relationship between high DAL and cancer incidence. However, all the studies reviewed have addressed this concern and considered the confounding effect of the factors in the DAL-cancer relationship analysis.

The exact mechanism connecting high DAL to the risk of cancer remains yet unclear. There are several potential hormonal and non-hormonal mechanistic pathways to demonstrate the long-term effect of diet-dependent acidosis on carcinogenesis as follows:

1) An acidosis diet May increase carcinogenesis by reduction of adiponectin secretion.

**Figure 4:**
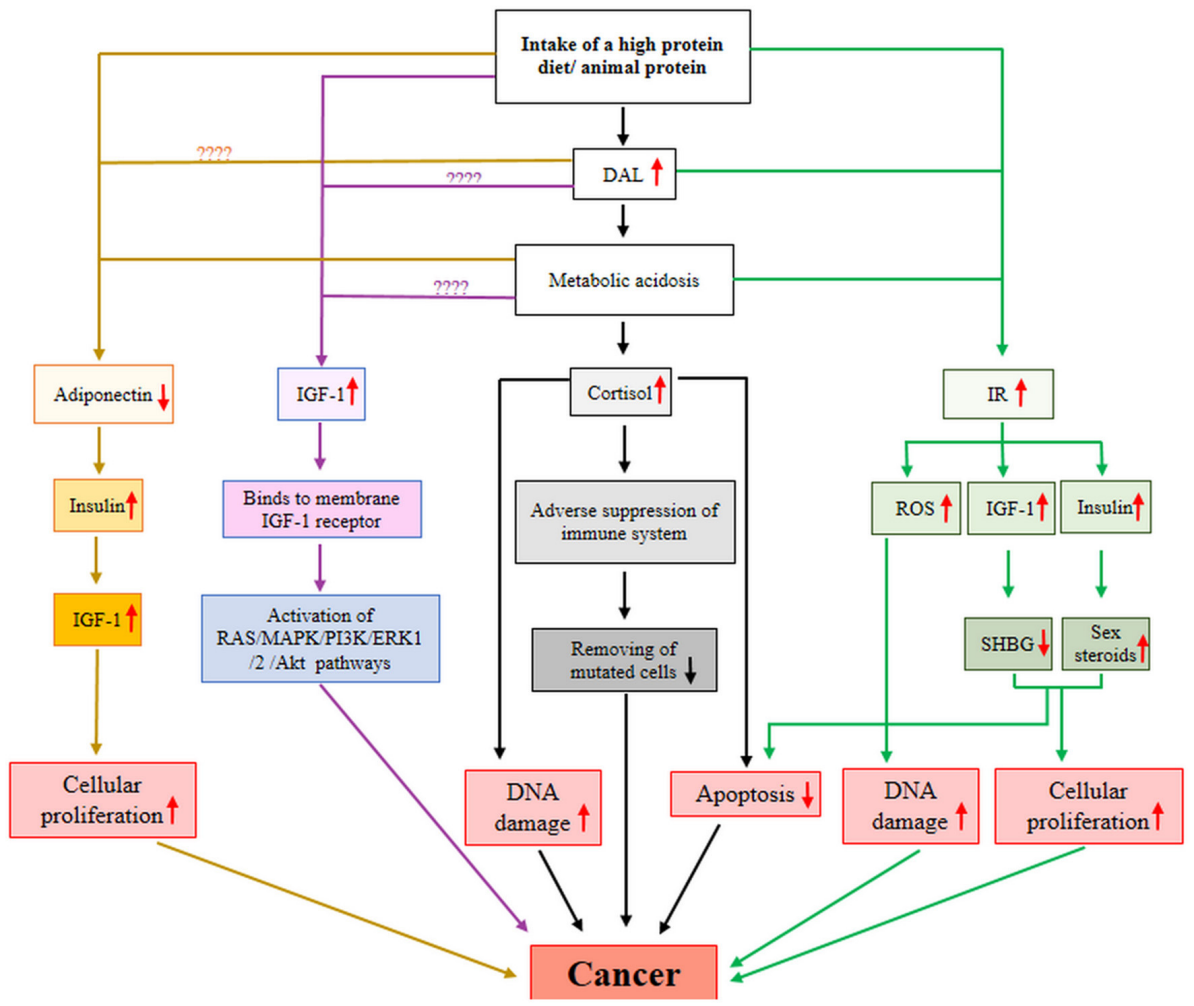
A possible mechanistic model for DAL-cancer relationship. AKt, protein kinase B; DAL, dietary acid load; ERK1, extracellular signal-regulated kinase 1; IGF-1, insulin-like growth factor-1; IR, insulin resistance; MAPK, mitogen-activated protein kinase; PI3K, Phosphoinositide 3-kinases; ROS, reactive oxygen species; SHBG, sex-hormone binding globulin.

Adiponectin is a 244-amino acid protein secreted mainly by adipocytes and act as an endogenous insulin sensitizer. Low circulating adiponectin level is supposed to have a critical role in the development and progression of multiple malignancies (39). As shown in Figure 4, low adiponectin level contributes to increased insulin level, which in turn, leads to elevated levels of bioavailable insulin-like growth factor (IGF)-1 (40). Insulin and IGF-1 induce cellular proliferation and prevent apoptosis and are therefore involved in carcinogenesis (40).

A diet's protein content and origin may contribute to adiponectin production. Yagi et al. found that a low-protein diet significantly elevated serum adiponectin level and also increased the amount of adiponectin secreted by adipocytes isolated from white adipose tissue (41). Also, Ceolin et al. in an animal model study showed that serum adiponectin level was higher in animals fed with a low protein diet than standard protein diet (42). The results of a study on older women participating in a resistance-based exercise program showed that women with a high protein diet had significantly higher adiponectin content compared to those with a high carbohydrate diet (43). The source of protein may be a reason for the discrepancy observed in the studies finding. According to evidence, consumption of animal protein may reduce the level of adiponectin. Chen et al. reported that serum adiponectin level was lower in rats fed animal-based protein diet than rats fed vegan protein-based diets (44). Moreover, a large body of evidence indicates a positive relationship between high adherence to plant-based diets such as the Mediterranean diet and serum adiponectin level (45, 46). Furthermore, endogenous metabolic acidosis, as an outcome of DAL, may also lead to a reduced level of adiponectin. Disthabanchong et al. in *in-vivo* and *in-vitro* studies showed that metabolic acidosis prevented adiponectin gene expression and reduced adiponectin serum levels (47). There was no study to show the effect/association of DAL on/with adiponectin level.

Taken all together, consumption of animal-based protein and metabolic acidosis state in the body may diminish the level of adiponectin, which in turn, increases the risk of cancer. Further research is required to evaluate the effect/association of the acidosis diet on/with the adiponectin level.

2) An acidosis diet May increase carcinogenesis by elevation of cortisol production.

Cortisol is a stress hormone that controls numerous processes throughout the body, such as metabolism and performance of the immune system. A growing body of evidence is suggesting a positive relationship between high cortisol levels and the progression of cancer (48–50). High cortisol concentrations adversely suppress the immune system and decrease its sufficiency in eliminating mutated cells (48). In addition, higher cortisol concentrations may contribute to the development of cancer by increasing DNA damage and apoptosis suppression (48) (Figure 4).

The amount of protein in a diet or an acidifying diet or metabolic acidosis condition in the body may enhance the production of cortisol. Slag et al. showed that consumption of a high protein diet contributed to the increased release of cortisol in healthy individuals (51). Also, Lemmens et al. reported that consumption of a high-protein meal increased cortisol levels in men and women (52). Esche et al. suggested that the presence of a moderate increase in diet-dependent acid load is adequate to increase glucocorticoids secretion and influence cortisol metabolism (53). Buehlmeier et al. showed that diet-dependent acidification/alkalization influenced glucocorticoids activity and metabolism, in healthy men (54). Perez et al. in a study on dogs reported that metabolic acidosis was associated with increased plasma cortisol levels of animals (55).

3) An acidosis diet May increase carcinogenesis by elevation of circulating IGF-1 level.

The insulin-like growth factor is a hormone with a critical role in the growth and mediates the anabolic effects of growth hormone or protein synthesis in muscle and skeletal tissues. Elevated circulating IGF-1 level promotes tumorigenesis, angiogenesis, and metastasis (56–58). IGF-1 stimulates several signaling pathways such as PI3K/Akt and MAPK through binding to its cell surface receptor and induces cancer cell proliferation, survival, and migration (57) (Figure 4).

Diet protein level or acidity or metabolic acidosis state of the body may elevate the production of IGF-1. An extensive body of studies have consistently indicated that intake of a high protein diet up-regulates the IGF-1 level. Schüler et al. reported that consumption of a high protein diet significantly increased IGF-1 levels in patients with type 2 diabetes (59). Giovannucci et al., in a study on 753 men, reported that men with high total protein intakes had a 25% greater plasma IGF-1 level than those with low protein intake (60). Drake et al. found that high protein intake was associated with high plasma IGF-1 level, in women older than 50 years (61). Morgan et al. showed that low protein intake was associated with a reduced level of IGF-1 in the population aged ≤ 65 years (62). Wan et al. showed that serum IGF-1 and liver IGF-1 mRNA levels were lower in pigs fed with low-protein than pigs fed with normal crude protein (63). Regarding the source of protein, Hoppe et al. reported that serum IGF-1 level was significantly associated with intakes of animal protein and milk, but not with the intake of vegetable protein or meat (64). Schüler et al. reported that both animal and plant protein intake lead to significant increases of IGF-1 level, which was unchanged by the various amino acids plant and animal protein composition, in participants with type 2 diabetes (59).

Concerning the association between DAL and IGF-1 concentration, research is too scarce. In a study, Lim et al. did not find any interaction effects between DAL and IGF-1 (65). Moreover, several relatively archaic studies have indicated that NH4Cl-induced metabolic acidosis reduces IGF-1 (66, 67). Additional researches to examine the association between DAL and dietary-induced metabolic acidosis with serum levels of IGF-1 are needed to better understand how high protein intake may affect IGF-1 level.

4) An acidosis diet May increase carcinogenesis by elevation of insulin resistance.

Insulin resistance (IR) is a pathological condition that presents when a disturbance occurs in the biological response to insulin. IR is well known to raise the risk of metabolic diseases such as cancer (68, 69). The possible mechanism for this association has fully been explained by Arcidiacono et al. (68). In brief, as shown in Figure 4, IR leads to hyperinsulinemia and enhancement of bioavailable IGF-1, which both of them prevent the hepatic production of sex-hormone binding globulin and induce ovarian production of sex steroids. Finally, these alterations stimulate cellular proliferation and prevent apoptosis (68). IR may contribute to carcinogenesis through impaired DNA due to excess production of reactive oxygen species (68).

Consumption of a high protein diet, DAL, and metabolic acidosis may impact IR level. Results of a systematic review and meta-analysis of randomized controlled trials showed that intake of a high-protein diet may reduce IR levels in patients with type 2 diabetes (70). Morenga et al., in an interventional study on overweight or obese women, found that insulin sensitivity reduced by 19.3% after intake of a diet relatively high in both protein and fiber compared with a standard diet (71). The source of protein is an important factor for the modifying of IR. Azemati et al. in a cross-sectional study on 548 participants showed that intake of total protein, animal protein, and the ratio of animal-to-plant protein intake were positively linked to IR, but plant protein was not (72). Adeva-Andany et al. in a review study discussed the contribution of animal protein intake on increased IR, in various population groups (73). Furthermore, Wojcik et al. in an animal model study showed that a high-protein casein diet (animal protein) had a minimal benefit in reduction of IR compared with a high-protein soy diet, or high-protein combined diet with animal and plant proteins (74).

Concerning the association between DAL and IR level, Lee et al., in a study on 5,406 participants, concluded that DAL was positively correlated to the development of IR (75). Also, Akter et al. in a study on 1732 workers found that high DAL was positively associated with IR (76). Endogenous metabolic acidosis is another DAL-related factor that may influence IR level. Williams et al. in a cross-sectional study found that individuals with IR had a higher level of fasting plasma lactate, a marker of metabolic acidosis (77). Bellasi et al. in a study on 145 patients with chronic kidney disease showed that rectification of metabolic acidosis ameliorates IR (78).

## Strengths and Limitations of the Study

The inclusion of several prospective cohort studies with large sample sizes, in the present review, enhances the power of the findings. All the studies, except one, used the standard FFQ method to assess food intake, and all the studies used two validated measurements of PRAL and NEAP for DAL assessment which make it possible to compare results among studies. Studying various types of cancer across the studies was a limitation of the present study, which may impact the comparability of the findings. All the included studies were observational and there was no intervention study.

## Conclusions

The findings indicate that higher DAL may be associated with a higher risk of cancer incidence across cancer types, study populations, both genders, both DAL assessment indicators (PRAL and NEAP), and also among both high- and low-risk age groups for cancer.

## Application of the Findings

This finding highlights that high DAL, which reveals the metabolic and nutritional status of an individual, may have long-term effects on human health. As a primary prevention strategy against cancer, the elevation of knowledge and attitudes of people at the community level, toward harms of diets with high acid load through training and advertising may navigate people to healthier dietary habits. Moreover, at the clinical level, the providing of dietary recommendations regarding foods with low DAL may be of help to prevent the development and progression of cancer.

## Suggestions for Future Research

Further evidence from interventional investigations is required to affirm findings from observational studies. Further researches are needed to evaluate: effect/association of the high DAL on/with the serum adiponectin and IGF-1 level, the relationship between dietary-induced metabolic acidosis with IGF-1, and the effect/association of high DAL on/with cellular proliferation, apoptosis, and signaling pathways involved in these events.

## Data Availability Statement

The original contributions presented in the study are included in the article/Supplementary Material, further inquiries can be directed to the corresponding author/s.

## Author Contributions

SK and MA contributed to the concept, design, interpretation of the data, and preparation of the manuscript. MK and VM contributed to the articles searching process, data extraction and analysis. SK is responsible for design, writing, and final content of the manuscript. All authors have read and approved the final version of the manuscript.

## Funding

This study was funded by Tabriz University of Medical Sciences, Tabriz , Iran (grant number 68371).

## Conflict of Interest

The authors declare that the research was conducted in the absence of any commercial or financial relationships that could be construed as a potential conflict of interest.

## Publisher's Note

All claims expressed in this article are solely those of the authors and do not necessarily represent those of their affiliated organizations, or those of the publisher, the editors and the reviewers. Any product that may be evaluated in this article, or claim that may be made by its manufacturer, is not guaranteed or endorsed by the publisher.
