# Supplementary material for: Association of High Dietary Acid Load With the Risk of Cancer: A Systematic Review and Meta-Analysis of Observational Studies
Source: Front Nutr. 2022 Mar 28;9:816797. doi: 10.3389/fnut.2022.816797 (PMC8997294; doi:10.3389/fnut.2022.816797)
Supplement: Supplementary file 1 [file Table_1.DOCX]

| **Supplementary Table 1.** Association between the dietary acid load and cancer: Method of the database search strategy using PubMed, SCOPUS, Google Scholar, Web of Science and Cochrane Library | | |
| --- | --- | --- |
| Database (Search  conducted up to  June 24, 2021) | Search terms^a^ | Number of studies searched |
| PubMed | #1 acid load[Title/Abstract] OR "dietary acid load"[Title/Abstract] OR "potential renal acid load"[Title/Abstract] OR "net endogenous acid production"[Title/Abstract]  #2 cancer[MeSH Terms]  #1 & #2 (acid load[Title/Abstract] OR "dietary acid load"[Title/Abstract] OR "potential renal acid load"[Title/Abstract] OR "net endogenous acid production"[Title/Abstract]) AND (cancer[MeSH Terms]) | 1505  3,492,961  65 |
| SCOPUS | ( TITLE-ABS-KEY ( "acid load" OR "dietary acid load" OR "potential renal acid load" OR "net endogenous acid production" ) AND TITLE-ABS-KEY ( cancer ) ) | 58 |
| Google Scholar | * allintitle: “acid load” "dietary acid load" "potential renal acid load"  with the exact phrase: “net endogenous acid production”  with at least one of the words: acid load OR "dietary acid load" OR "potential renal acid load" OR "net endogenous acid production" | 357 |
| Web of Science | TITLE: (acid load OR "dietary acid load" OR "potential renal acid load" OR "net endogenous acid production") AND TITLE: (cancer) | 221 |
| Cochrane library | Cochrane Library  #1 "acid load" OR "dietary acid load" OR "potential renal acid load" OR "net endogenous acid production" in Title Abstract Keyword  #2 cancer  #1 & #2 “acid load” OR "dietary acid load" OR "potential renal acid load" OR "net endogenous acid production" in Title Abstract Keyword AND "Cancer" in Title Abstract Keyword | 183  167526  4 |
| Total |  | 705 |
| ^a^Searches were limited to original articles, and studies published in the English language using the appropriate filters and/or search terms depending on the database. | | |
