# Supplementary material for: Association of High Dietary Acid Load With the Risk of Cancer: A Systematic Review and Meta-Analysis of Observational Studies
Source: Front Nutr. 2022 Mar 28;9:816797. doi: 10.3389/fnut.2022.816797 (PMC8997294; doi:10.3389/fnut.2022.816797)
Supplement: Supplementary file 2 [file Table_2.DOCX]

| **Supplementary Table 2.** Newcastle-Ottawa scale for quality assessment of seven included case-control studies assessing the relationship of dietary acid load and cancer (each asterisk represents if individual criterion within the subsection was fulfilled) | | | | | | | | |
| --- | --- | --- | --- | --- | --- | --- | --- | --- |
| Quality assessment criteria | Acceptable (*) | Jafari et al. 2020 | Mehranfar et al. 2020 | Mousavi et al. 2019 | Ronco et al. 2020 | Ronco et al. 2021 | Ronco et al. 2021 | Safabakhsh et al. 2020 |
| **Selection** | | | | | | | | |
| Is the case definition adequate? | yes, with independent validation | * | * | * | * | * | * | * |
|  | yes, eg record linkage or based on self-reports |  |  |  |  |  |  |  |
| Representativeness of the cases | consecutive or obviously representative series of cases | * | * | * | * | * | * | * |
| Selection of Controls | community controls |  |  |  |  |  |  |  |
|  | hospital controls | * | * | * | * | * | * | * |
| Definition of Controls | no history of disease (endpoint) | * | * | * | * | * | * | * |
| **Comparability** | | | | | | | | |
| Comparability of cohorts on the basis of the design or analysis controlled for confounders | The study controls for age and BMI | - | * | * | * | * | * | * |
|  | Study controls for other factors | * | * | * | * | * | * | * |
| **Exposure** | | | | | | | | |
| Ascertainment of exposure | secure record |  |  |  |  |  |  |  |
|  | structured interview | * | * | * | * | * | * | * |
| Same method of ascertainment for cases and controls | yes | * | * | * | * | * | * | * |
| Non-Response rate | same rate for both groups | * | * | - | - | * | * | * |
| **Overall Quality Score (Maximum = 9)** | | 8 | 9 | 8 | 8 | 9 | 9 | 9 |
