# Supplementary material for: Association of High Dietary Acid Load With the Risk of Cancer: A Systematic Review and Meta-Analysis of Observational Studies
Source: Front Nutr. 2022 Mar 28;9:816797. doi: 10.3389/fnut.2022.816797 (PMC8997294; doi:10.3389/fnut.2022.816797)
Supplement: Supplementary file 3 [file Table_3.DOCX]

| **Supplementary Table 3.** Newcastle-Ottawa scale for quality assessment of three included cohort studies assessing the relationship of dietary acid load and cancer (each asterisk represents if individual criterion within the subsection was fulfilled) | | | | |
| --- | --- | --- | --- | --- |
| Quality assessment criteria | Acceptable (*) | Park et al. 2019 | Shi et al. 2021 | Wu et al.  2020 |
| **Selection** | | | | |
| Representativeness of exposed cohort? | Truly representative | * | * | * |
|  | Somewhat representative |  |  |  |
| Selection of the non-exposed cohort? | Drawn from same community as the exposed cohort | * | * | * |
| Ascertainment of exposure? | Secured records |  |  |  |
|  | Structured interview | * | * | * |
| Demonstration that outcome of interest was not present at start of study? | yes | * | * | $*^{a}$ |
| **Comparability** | | | | |
| Comparability of cohorts on the basis of the design or analysis controlled for confounders | The study controls for age and BMI | * | * | * |
|  | Study controls for other factors | * | * | * |
| **Outcome** | | | | |
| Assessment of outcome? | Independent blind assessment | * | * | * |
|  | Record linkage |  |  |  |
| Was follow-up long enough for outcomes to occur | yes | * | * | * |
| Adequacy of follow-up of cohorts | Complete follow up- all subject accounted for | * | * | * |
|  | Subjects lost to follow up unlikely to introduce bias- number lost less than or equal to 20% or description of those lost suggested no different from those followed. | - | - | - |
| **Overall Quality Score (Maximum = 9)** | | 9 | 9 | 9 |
| a: Participants in the study were people with breast cancer who had recovered and were screened for recurrence. | | | | |
