# Supplementary figures and images for: Association of High Dietary Acid Load With the Risk of Cancer: A Systematic Review and Meta-Analysis of Observational Studies
Source: Front Nutr. 2022 Mar 28;9:816797. doi: 10.3389/fnut.2022.816797 (PMC8997294; doi:10.3389/fnut.2022.816797)

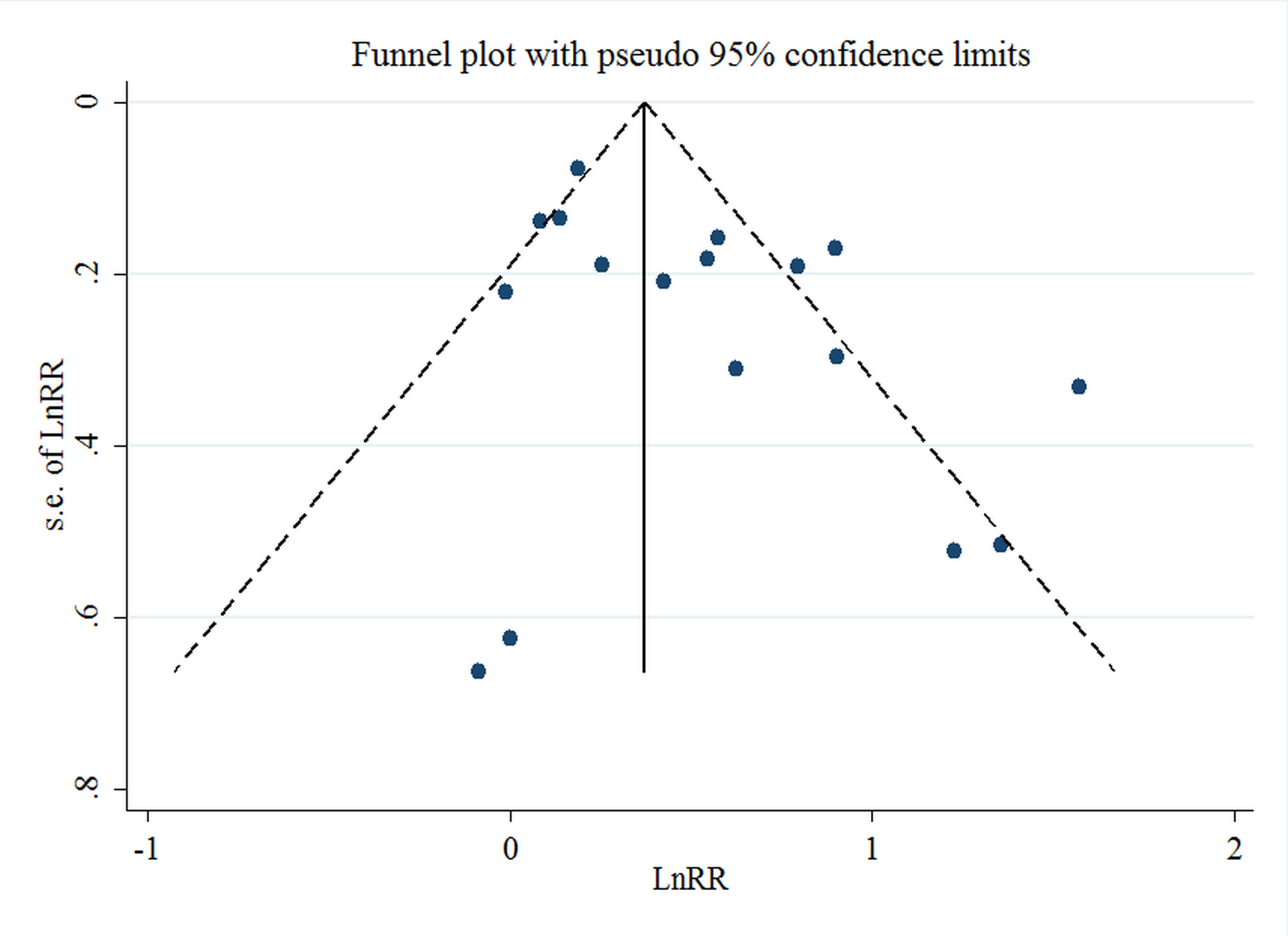

Supplement: Supplementary file 5 [file Image_1.TIF]

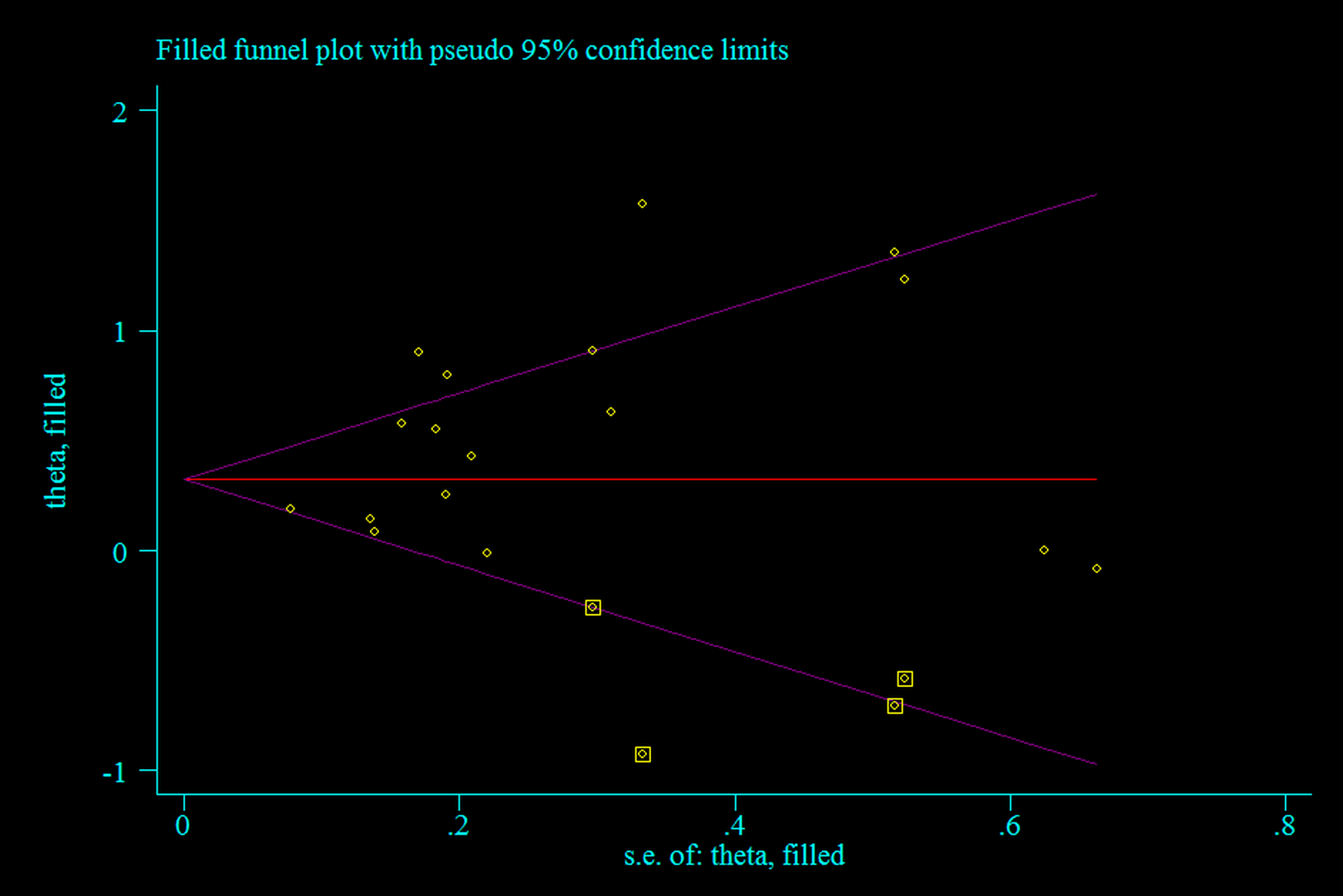

Supplement: Supplementary file 6 [file Image_2.TIF]
